# Acquired immunity mechanisms in sheep infected with *Nematodirus oiratianus*: key genes and host responses

**DOI:** 10.3389/fvets.2025.1643786

**Published:** 2025-09-04

**Authors:** Bin Hou, Rina Su, Dalai Suyala, Li Xu, Ruiyuan Zhao, Puguo Hao, Yali Wang, Fei Mao, Bo Wu, Xuedong Ding, Runqing Han, Xuesong Peng, Risu Na, Yihong Hao, Qimuge Bainuwen, Xiaojia Mu, Surong Hasi

**Affiliations:** ^1^Ordos Vocational College of Eco-Environment, Ordos, China; ^2^Baotou Light Industry Vocational Technical College, Baotou, China; ^3^Inner Mongolia Hua’ao Kexing Biotechnology Co., Ltd, Ordos, China; ^4^College of Animal Science, Inner Mongolia Agricultural University, Hohhot, China; ^5^Key Laboratory of Clinical Diagnosis and Treatment Technology in Animal Diseases, Ministry of Agriculture, College of Veterinary Medicine, Inner Mongolia Agricultural University, Hohhot, China

**Keywords:** sheep, *Nematodirus oiratianus*, gastrointestinal nematode control, transcriptomic, proteomic

## Abstract

**Background:**

Controlling gastrointestinal nematode infections poses a major challenge in intensive farming, worsened by rising anthelmintic resistance. Developing innovative control strategies is critical. Sheep acquired immunity to *Nematodirus oiratianus* offers a valuable model for dissecting host-nematode interactions, though underlying immune mechanisms remain unclear.

**Methods:**

This study employed multi-omics analyses (transcriptome and proteome) of lamb duodenum across infection stages, validated by RT-qPCR, iELISA, histopathological staining (HE), and electron microscopy.

**Results and conclusions:**

Infection triggered early immune activation mediated by intestinal epithelial cells. Key differentially expressed genes included *CLDN18*, *CCL19*, *FGB*, and *Muc5ac*, potentially linking to parasite expulsion. Early-stage pathway enrichment in cancer, chemokine signaling, and phagosome function enhanced intestinal barrier integrity and immune cell recruitment. Late-stage upregulation of *CCL* chemokines directly impacted nematode survival. Intestinal villi shedding was also correlated with parasite clearance. These findings unravel potential mechanisms of acquired immunity against *Nematodirus oiratianus*, highlighting novel therapeutic targets (e.g., epithelial barrier regulators, chemokine networks) and vaccine candidates for nematode control in livestock.

## 1 Introduction

*Nematodirus oiratianus* (*N. oiratianus*), belonging to the genus *N. oiratianus* of the family Trichostrongylidae, is a highly pathogenic gastrointestinal nematode that parasitizes the small intestine of ruminants such as cattle, sheep, and camels. The adult worms are similar in appearance to *Haemonchus contortus* (*H. contortus*), but the anterior part of the worm body is slender while the posterior part is wider, hence the name “*Nematodirus*” (thin-necked nematode). *Nematodirus* was first described in the United Kingdom in 1951, it likely originated in the Arctic region and spread through animal migration. Originally described in British sheep in 1951 (1), its impact continues to pose a substantial threat to lamb health across numerous temperate regions worldwide. Surveys have reported a prevalence of *Nematodirus* spp. infection at 40% among wild ungulates in the Hirpora Wildlife Sanctuary, Kashmir, India (2), and at 16.8% among dairy goats in Greece (3). Despite this, it is often overlooked, even though it ranks as the second most common nematode infection within the Trichostrongylidae family, following *Haemonchus contortus*. *Nematodirus* spp. is a soil-transmitted gastrointestinal nematode that does not rely on an intermediate host and is distributed from the Arctic to South Africa. Its egg hatching requires a freezing stimulus to enhance hatching rates (4), which indirectly suggests its possible origin from deer inhabiting cold Arctic regions. With the expansion of livestock farming and other cultural practices (5), this nematode has gradually gained prominence globally, leading to its widespread distribution (6–10). Adult worms predominantly inhabit the anterior segment of the small intestine. Females produce relatively few eggs per day, yet their eggs are among the largest in the Trichostrongylidae family, comparable to those of *Marshallagia* spp. (11). Freshly excreted eggs contain 6–8 oocytes, with larvae developing into infective forms within the eggs.

Unlike other nematodes of the family Trichostrongylidae, sheep exhibit acquired immunity to nematodes of the *Nematodirus* spp. However, the magnitude of this protective immune response and the extent of infection are also influenced by additional factors, including parasite prevalence, host susceptibility, and nutritional status of the host. The most frequently reported species is *Nematodirus battus*, but other species within the *Nematodirus* genus share similar characteristics. This acquired immunity enables the elimination of nematodes from the intestine within 24–34 days post-infection (12, 13). The development of this acquired immune response has been demonstrated to rely on both the size of the initial parasitic challenge and the individual immunological response of the animal (14). This appears to be a survival strategy for *Nematodirus* spp., maintaining a high infection rate coupled with low mortality. This dynamic equilibrium is advantageous for *Nematodirus* spp., facilitating easier host finding throughout their life cycle. Lambs have been observed to mount a specific IgM and IgG antibody response approximately 18 days post-infection (15), accompanied by a significant increase in eosinophils and mast cells in the intestinal region where adult worms establish themselves. The development of infective larvae of *Nematodirus* spp. in the anterior segment of the host’s small intestine involves the secretion of various proteins, some of which act as potent immunomodulators, inducing Th2-type immune responses in the intestines, ultimately leading to worm expulsion (13). Crystalline formations have been detected in worms following high infection levels, which, located at the posterior end of the intestine, obstruct the intestinal-rectal junction, resulting in fluid accumulation behind the blockage and degeneration of the reproductive organs (16, 17). Some genes, proteins, and metabolites during parasite infection and host resistance to infection play a crucial role in parasite–host interactions, and these are particularly important for maintaining infection and modulating host immune responses, in addition to some physiological functions. Therefore, they are also considered as candidates for drug development or vaccine development. It has been shown that *Nematodirus* spp. infection reduces the activity of several intestinal enzymes, including leucine aminopeptidase, alkaline phosphatase, and disaccharidases involved in protein and sugar digestion (18). The exact mechanism by which the intestine changes in response to helminth infections is also unknown, with many studies demonstrating that the intestine can change the pH of the intestinal lumen, the intestinal flora, and the shedding of intestinal villi to resist parasitic infection (17, 19–21).

Acquired immunity to this phenomenon has been reported long ago, but our current research lacks sufficient insight into the intricacies of parasite–host interactions. In contrast to other nematodes, the infective larvae of *Nematodirus* spp. fully develop in eggs and do not ingest other bacteria or nutrients. Therefore, their establishment of infection may be simpler, and host recognition of their infection more precise than that of other nematodes. To delve into the mechanism of this phenomenon and analyze the changes occurring in the host during dynamic parasite infection, we scrutinized the mRNA, protein, and alterations in the sheep duodenum at various infection periods following exposure to *Nematodirus oiratianus*. By doing so, we aimed to determine the mechanisms contributing to the occurrence of acquired immunity. The results of this study provide fresh insights into our understanding of parasite–host interactions and unveil novel drug or vaccine targets with potential roles in these interactions.

## 2 Materials and methods

### 2.1 Institutional review board statement

Animal procedures were performed in accordance with the National Standard Guideline for Ethical Review of Animal Welfare (GB/T 35892-2018) and approved by the Animal Care and Use Committee of Inner Mongolia Agricultural University. All efforts are made to take the least painful approach to animal handling and to avoid or reduce stress, pain and injury to the animal used in the experiment.

### 2.2 Sample collection and grouping

The *Nematodirus oiratianus* strain was isolated from lambs naturally grazing in Wushen Banner, Ordos (Latitude: 38.604136, Longitude: 108.817607), and confirmed as *Nematodirus oiratianus* through morphological examination of domestic nematodes and ITS-2 analysis (22). Following several generations of purification and culture, the strain was maintained in lambs. Fresh fecal samples were regularly collected using the sugar-saline floating method to obtain clean eggs, which were then repeatedly floated, cleaned by centrifugation, and cultured in distilled water at 27 °C for approximately 15 days to develop into infective larvae before being preserved for future use. Five Ordos fine wool sheep lambs, all of similar body condition and aged 3 months (weighing 15 kg ± 2 kg), were chosen as test subjects and underwent deworming with ivermectin, closantel, and sulfaquinoxaline prior to experimentation. Fecal samples were collected 7 days post-deworming and examined using the McMaster technique to confirm the absence of specific parasitic infections. The animals were housed in a controlled environment and provided with clean water and fecal-contamination-free forage (23).

After 15 days of acclimatization under uniform conditions, 20,000 *Nematodirus oiratianus* infective larvae were orally administered to each lamb. The third day post-infection constituted the infective L3 group, the seventh day the infective L4 group, the tenth day the L5 group, and the twenty-fifth day the infective adult (AD) group (24–26). A control group received an equivalent volume of distilled water. Each group was replicated three times. At 3, 7, 10, and 20 days post-infection, the anterior one-third segment of the duodenum was dissected following euthanasia via sodium pentobarbital injection. The contents were rinsed with pre-chilled PBS, and all collected samples were snap-frozen in liquid nitrogen and stored at −80 °C.

### 2.3 Transcriptomics

#### 2.3.1 RNA extraction and sequencing

Transfer the appropriate duodenal tissue to the corresponding grinding tube, then add 1.5 mL of TRIzol lysis buffer. Subsequently, total RNA was individually extracted from each sample using a mirVana miRNA Isolation Kit (BGI, Beijing, China) following the manufacturer’s protocol. The integrity of the RNA was assessed using an Agilent 2100 Bioanalyzer (Agilent Technologies, Santa Clara, CA, USA). Samples with an RNA integrity number (RIN) of ≥7 were selected for further analysis.

Libraries were constructed using a DNF-471 Standard Sensitivity RNA Analysis Kit (BGI, Beijing, China) as per the manufacturer’s instructions. These libraries were then sequenced on a DNBSEQ sequencing platform (DNBSEQ-T7RS, BGI, Beijing, China), producing 150 bp paired-end reads.

#### 2.3.2 Sequence filtering, functional annotation, and analysis of differentially expressed genes

The initial sequencing data includes reads of subpar quality, splice contamination, and a notable proportion of ambiguous base N. To ensure the reliability of our analyses, these problematic reads are eliminated prior to further processing. Following this data refinement step, we employ HISAT for the alignment of the clean reads to the reference genome sequence, which in this study is the Ovisaries genome (reference genome version: GCF016772045.1ARS-UIRamb_v2.0).

Identification of differentially expressed genes (DEGs) within each experimental group (comprising three replicates per group) is conducted using functions from the DESeq R package, specifically estimateSizeFactors and nbinomTest. A gene exhibiting a *p*-value < 0.05 and a log2foldchange ≥ 1 or ≤ −1 is deemed differentially expressed (27, 28). These differential genes undergo functional classification based on KEGG Pathway annotation criteria. Enrichment analysis is carried out via the phyper function in R software to compute the *p*-value, subsequently corrected to obtain the *Q*-value through FDR correction. Functions with a *Q*-value of ≤ 0.05 are considered significantly enriched.

Similarly, based on the GO annotation outcomes and the standard classification, the differentially expressed genes are functionally categorized. Enrichment analysis is once again performed using the phyper function in R software to calculate the *p*-value, which is then subjected to FDR correction to derive the *Q*-value. Typically, functions associated with a *Q*-value of ≤ 0.05 are regarded as significantly.

### 2.4 Quantitative proteomics (4D-DIA)

#### 2.4.1 Protein extraction, quantization, and SDS-PAGE electrophoresis

The sample was carefully weighed and then transferred to a 2 mL centrifuge tube. Two 5 mm magnetic beads were added, along with 1XCocktail (protease inhibitor) containing SDS (sodium dodecyl sulfate) and EDTA (diethylaminetetraacetic acid) at the final concentration. The beads underwent lysis using an automated grinder, followed by centrifugation at 25,000*g* * 4 °C for 15 min, and the supernatant was carefully removed. To this, DTT (dithiothreitol) was added at a final concentration of 10 mM, and the mixture was bathed for 30 min at 37 °C. Subsequently, IAM (iodoacetamide) was introduced at a final concentration of 55 mM and left in a dark room for 45 min.

To precipitate the proteins, 5 times the volume of pre-cooled acetone was added, and the solution was placed in a refrigerator at −20 °C for 2 h. Following this, centrifugation at 25,000*g* * 4 °C for 15 min was performed, and the supernatant was discarded. The precipitate was air-dried, and an appropriate amount of SDS-free protein lysis solution was added. An automatic grinder was employed to facilitate protein lysis. After another round of centrifugation at 25,000*g* * 4 °C for 15 min, the resulting supernatant was considered the protein solution.

The protein concentration was determined using the Bradford quantification method, and the proteins were stored at −80 °C. In addition, 7 μg samples underwent 12% SDS-PAGE, followed by visualization and scanning according to Candiano et al. (29).

#### 2.4.2 Protease digestion and high pH RP separation

Each sample comprised 100 μg of protein solution, to which 2.5 μg of Trypsin enzyme was added at a ratio of 40:1 (protein:enzyme). This mixture was enzymatically digested at 37 °C for 4 h, and the resulting peptides were desalted using a Strata X column and then vacuum dried. Equal amounts of peptides from all samples were mixed, diluted with mobile phase A (5% ACN, pH 9.8), and injected into the sample. The samples were separated in the liquid phase using a Shimadzu LC-20 AD liquid phase system with a 5 μm 4.6 × 250 mm Gemini C18 column. The gradient elution was conducted at a flow rate of 1 mL/min, starting with 5% mobile phase B (95% ACN, pH 9.8) for 10 min, followed by a linear increase from 5 to 35% mobile phase B over 40 min, a further increase to 95% mobile phase B over 1 min, maintaining mobile phase B for 3 min, and finally re-equilibration with 5% mobile phase B for 10 min. Elution peaks were monitored at 214 nm and collected at a rate of one per minute. The collected samples were combined based on the chromatographic elution peaks to yield 10 fractions, which were subsequently freeze-dried.

#### 2.4.3 DDA library building and DIA quantification (Nano-LC–MS/MS)

The desiccated peptide samples were reconstituted with mobile phase A (100% H_2_O, 0.1% FA), followed by centrifugation at 20,000*g* for 10 min. The resulting supernatant was introduced into the sample. The separation process was carried out using Bruker’s nanoElute system. Initially, the sample underwent enrichment and desalting on a trap column, and subsequently, it was separated in a series with a self-loading C18 column (75 μm I.D., 1.8 μm column material particle size, approximately 25 cm column length) at a flow rate of 300 nL/min. The separation was guided by a well-designed gradient: at 0 min, 2% mobile phase B (100% ACN, 0.1% FA); from 0 to 45 min, mobile phase B linearly increased from 2 to 22%; from 45 to 50 min, it further increased from 22 to 35%; then, from 50 to 55 min, mobile phase B rose from 35 to 80%; finally, at 55–60 min, it remained at 80% mobile phase B.

The liquid-phase-separated peptides were directly interfaced with the mass spectrometer. These peptides, separated in the liquid phase, underwent ionization through a CSI nano-liter source and were subsequently directed to the tandem mass spectrometer, timsTOF Pro, for both Data Dependent Acquisition (DDA) and Data Independent Acquisition Data Independent Acquisition (DIA) mode detection.

### 2.5 RT-qPCR validation

Based on the sequences of *CLDN18*, *CCL19*, *STAT4*, *MUC5AC*, and *FGB* obtained from the transcriptome, primers were designed using Primer 3, followed by fluorescence quantitative PCR. The reaction program was as follows: 95 °C for 30 s for denaturation (Stage 1), followed by denaturation at 95 °C for 15 s, annealing/extension at 60 °C for 30 s (Stage 2), and a gradual temperature increase from 65 °C to 95 °C at a rate of 0.5 °C per cycle with fluorescence signal collection at each increment (Stage 3). The results were analyzed using the ^ΔΔ^CT method. MiniBEST Universal Genomic DNA Extraction Kit Ver.5.0 (TaKaRa, 9765), SweScript All-in-One RT SuperMix for qPCR (TaKaRa, G3337), 2 × Universal Blue SYBR Green qPCR Master Mix (TaKaRa, G3326), and RNAlater (Ambion, AM7020) were all purchased from TAKARA. (Primer information has been added in the appendix).

### 2.6 Immunoglobulin and cytokine assays

Serum: After 3, 7, 10, and 20 days post-infection, blood was collected from the jugular vein of lambs. The blood was left at 4 °C overnight and then centrifuged at 2,000 rpm for 20 min. The supernatant was collected and stored at −20 °C for further analysis.

The main indicators for the determination of immunoglobulins and cytokines in the serum include IgA, IgM, IL-2, IL-5, IL-6, IL-10, INF-γ, and TNF-β. Quantitative determination was performed using a sandwich ELISA method with double antibodies. The specific procedures for the determination of the above indicators were conducted according to the instructions provided with the reagent kits.

### 2.7 Hematoxylin eosin staining for assessment of damage to the duodenum of lambs by infection with the *Nematodirus oiratianus*

According to the HE staining procedure, the middle section of the fresh lamb duodenum at different stages of infection was immersed in 4% paraformaldehyde and fixed overnight at room temperature. The duodenal tissue was removed from the fixative and placed in a fume hood. Using a surgical blade, the targeted tissue was trimmed and flattened. The trimmed tissue and corresponding labels were placed in a dehydration box. The dehydration box was placed in a dehydrator, and dehydration was carried out using a gradient of alcohol. This included 75% ethanol for 4 h, 85% ethanol for 2 h, 90% ethanol for 2 h, 95% ethanol for 1 h, absolute ethanol I for 30 min, absolute ethanol II for 30 min, alcohol benzene for 5–10 min, xylene I for 5–10 min, xylene II for 5–10 min, melting paraffin I at 65 °C for 1 h, melting paraffin II at 65 °C for 1 h, and melting paraffin III at 65 °C for 1 h. Embedding: The tissue soaked in wax was embedded in an embedding machine. First, melted wax was placed into the embedding mold. Before the wax solidified, the tissue was removed from the dehydration box, placed into the embedding mold according to the embedding surface requirements, and labeled accordingly. The embedding mold was cooled on a −20 °C freezing stage. Once the wax solidified, the wax block was removed from the embedding mold and trimmed. Sectioning: The trimmed wax block was cooled on a −20 °C freezing stage and then placed in a paraffin microtome for sectioning at a thickness of 4 μm. The sections were floated on a water bath at 40 °C to flatten the tissue, then picked up onto glass slides. The slides were placed in a 60 °C oven to dry after water evaporation and paraffin melting. Once dried, they were stored at room temperature for future use. Hematoxylin and eosin (HE) staining: Sections were stained in hematoxylin for 3–5 min, rinsed in tap water, differentiated, rinsed again in tap water, counterstained in eosin, and then rinsed under running water. Eosin staining: Sections were sequentially dehydrated in 85 and 95% gradient ethanol for 5 min each, then stained in eosin solution for 5 min. Dehydration and sealing: Sections were successively dehydrated in absolute ethanol I for 5 min, absolute ethanol II for 5 min, absolute ethanol III for 5 min, xylene I for 5 min, and xylene II for 5 min. After transparency, they were sealed with neutral resin. Microscopic examination and image acquisition analysis.

### 2.8 Scanning electron microscopic assessment of damage to the duodenum of lambs by infection with the *Nematodirus oiratianus*

Sample fixation: Fresh duodenum samples from lambs at different stages of *N. oiratianus* infection were gently rinsed with pre-cooled PBS to remove blood stains and intestinal contents from the intestinal surface. They were then quickly immersed in electron microscopy fixative and fixed at room temperature for 2 h before being transferred to 4 °C for storage. Post-fixation: The fixed samples were washed three times with 0.1 M phosphate buffer (pH 7.4) for 15 min each time. They were then fixed in 1% osmium tetroxide prepared in 0.1 M phosphate buffer (pH 7.4) at room temperature in the dark for 1–2 h. Afterward, the samples were washed three times with 0.1 M phosphate buffer (pH 7.4) for 15 min each time. Dehydration: The tissues were dehydrated sequentially in 30–50%, 70–80%, 90–95%, 100–100% ethanol for 15 min each, followed by immersion in isoamyl acetate for 15 min. Drying: The samples were dried in a critical point dryer. Sample conductive coating: The samples were tightly attached to a double-sided conductive carbon adhesive and placed on the sample stage of an ion sputtering instrument for gold sputtering for about 30 s. They were then observed and imaged under a scanning electron microscope.

## 3 Results

Headings, we utilized the DNBSEQ-T7RS platform to acquire 741.88 million raw reads from cDNA libraries across various infection periods, yielding over 670.02 million reads post-processing. On average, each sample contributed 6.70 gigabytes of data, resulting in the detection of a total of 18,605 genes. Following the acquisition of clean reads, we employed HISAT to align them with the reference genome sequence, achieving a clean read alignment rate of 95.9% to the reference genome and 90.17% to unique positions within it. The high correlation coefficient of single gene expression levels across different samples, nearing 1, indicates a strong similarity in expression patterns among samples.

Additionally, within this project, mass spectrometry data were gathered from 24 samples using a TimsTOF Pro instrument in DIA mode, resulting in the quantification of 751,740 peptides and 106,116 proteins.

### 3.1 Differentially expressed genes (DEGs) and differentially expressed proteins (DEPs)

**Figure 1:**
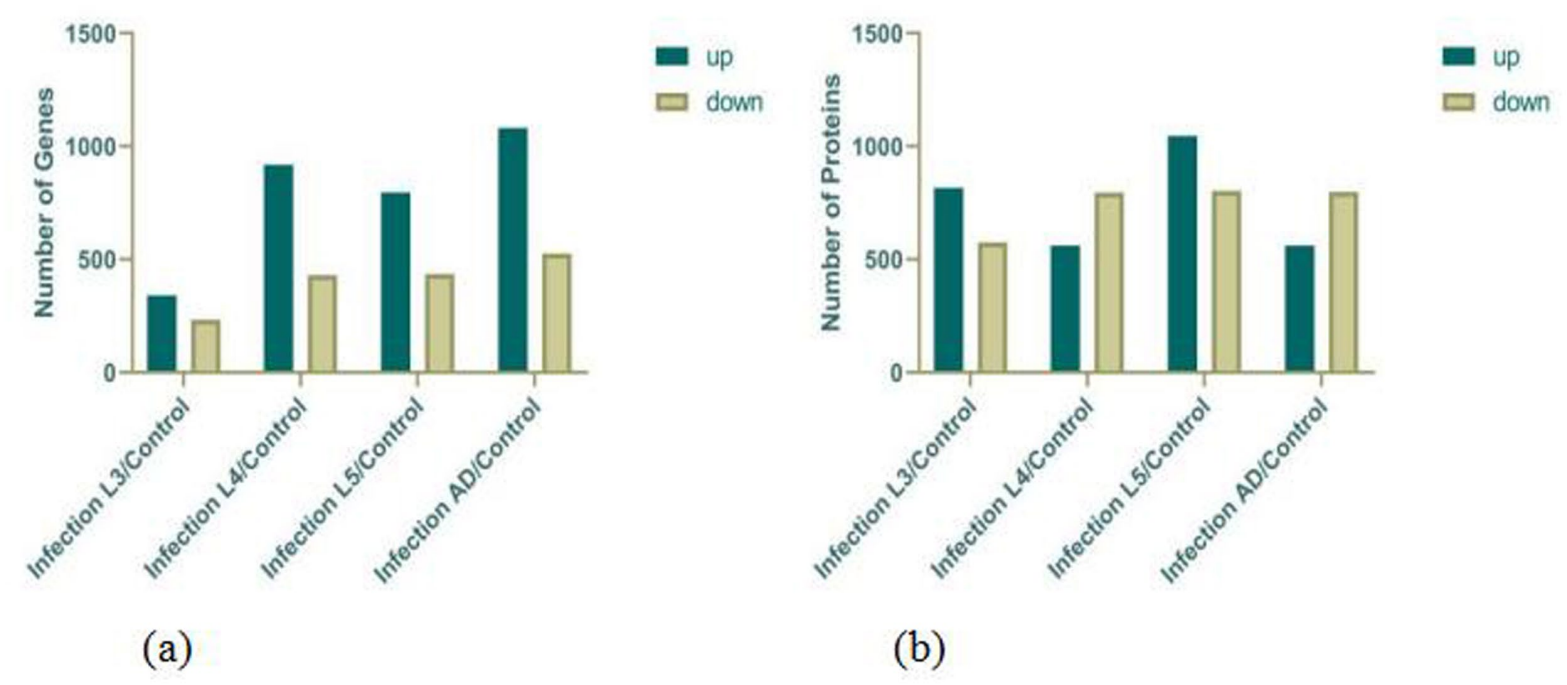
Statistics on the DEGs **(a)** and DEPs **(b)**.

According to the gene expression profiles of each group of samples, differential gene analysis was performed using DESeq2, with the criteria for selecting differentially expressed genes set as *Q* value ≤0.05 and |log2FC| ≥ 1. The results, as indicated in the Figure 1, show the following: Clustering analysis of the above DEGs (differentially expressed genes) revealed that when comparing free-living L1–L3 stages with eggs, upregulated genes were primarily associated with actin binding, symporter activity, and neuropeptide hormone activity, while downregulated genes were mainly related to carbohydrate binding. In contrast, when parasitic L4–L5 stages were compared with eggs, upregulated genes were predominantly involved in metal ion binding, lipid binding, and phospholipid metabolic process. For adult male and female worms versus eggs, upregulated genes were mainly linked to adherens junction, metal ion binding, lipid transporter activity, nutrient reservoir activity, lipid catabolic process, and chitin binding, while downregulated genes were primarily associated with arachidonic acid secretion. These findings indicate that during egg development, upregulated genes are predominantly related to the development of the muscular and nervous systems, whereas during the parasitic stage, they are mainly involved in biological processes such as nutrient metabolism and reproduction.

Based on the protein expression profiles of each group of samples, differentially expressed proteins were screened using Fold change >2 and *p*-value <0.05 as the criteria. The results, as indicated in the figure, show the following: Clustering analysis of the above DEPs (differentially expressed proteins) revealed that when comparing free-living L1–L3 stages with eggs, upregulated proteins were primarily associated with cuticle development, molting cycle, collagen and cuticulin-based cuticle, actin filament organization, myosin filament assembly, and locomotory behavior, while downregulated proteins were mainly related to negative regulation of translation, anaerobic respiration, lipid storage, regulation of mitotic cell cycle, and carbohydrate metabolic process. This reflects biological processes such as molting during larval development and energy consumption reduction in infective larvae. In contrast, when parasitic L4–L5 stages were compared with eggs, upregulated proteins were predominantly involved in muscle organ development, glycolytic process, nervous system process, and metabolic process. For adult male and female worms versus eggs, upregulated proteins were mainly linked to mitotic cytokinesis, innate immune response, carbohydrate metabolic process, lipid catabolic process, and chitin binding, while downregulated proteins were primarily associated with dauer larval development (Figure 1).

### 3.2 Gene ontology (GO) analysis of DEGs

**Figure 2:**
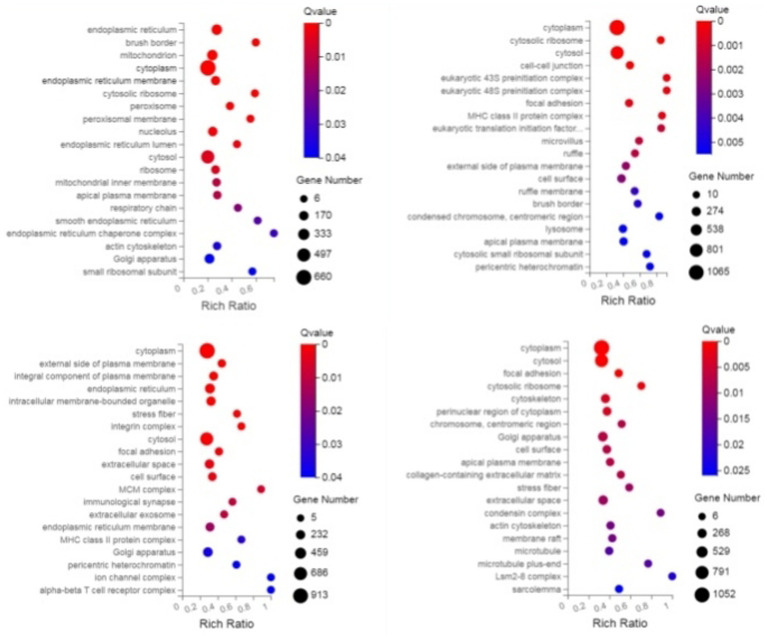
GO enrichment bubble plot of DEGs (sequentially infection L3 group, infection L4 group, infection adult group vs. control group).

Through GO enrichment analysis, when compared with eggs, DEGs at each developmental stage were predominantly enriched in biological processes related to the integral component of membrane and metal ion binding, which may be associated with the blood-feeding biological characteristics of *N. oiratianus*. Additionally, in L3 stage, nucleus and DNA binding were significantly enriched, while in L4 stage, nucleus, RNA binding, and DNA binding were significantly enriched. Proteins can regulate various cellular functions through interactions with nucleic acids (DNA and RNA). The significant enrichment of DNA binding and RNA binding reflects the rapid transition of *N. oiratianus* from the free-living stage to the parasitic stage, where transcriptional regulation, alternative splicing, and modification of DEGs control the development of the nematode. In the female and male worm groups, the extracellular region was significantly enriched, indicating biological processes such as signal transduction with the host intestinal environment, cell adhesion, extracellular matrix formation, cell motility, and tissue structure maintenance (Figure 2).

### 3.3 KEGG analysis of DEGs

**Figure 3:**
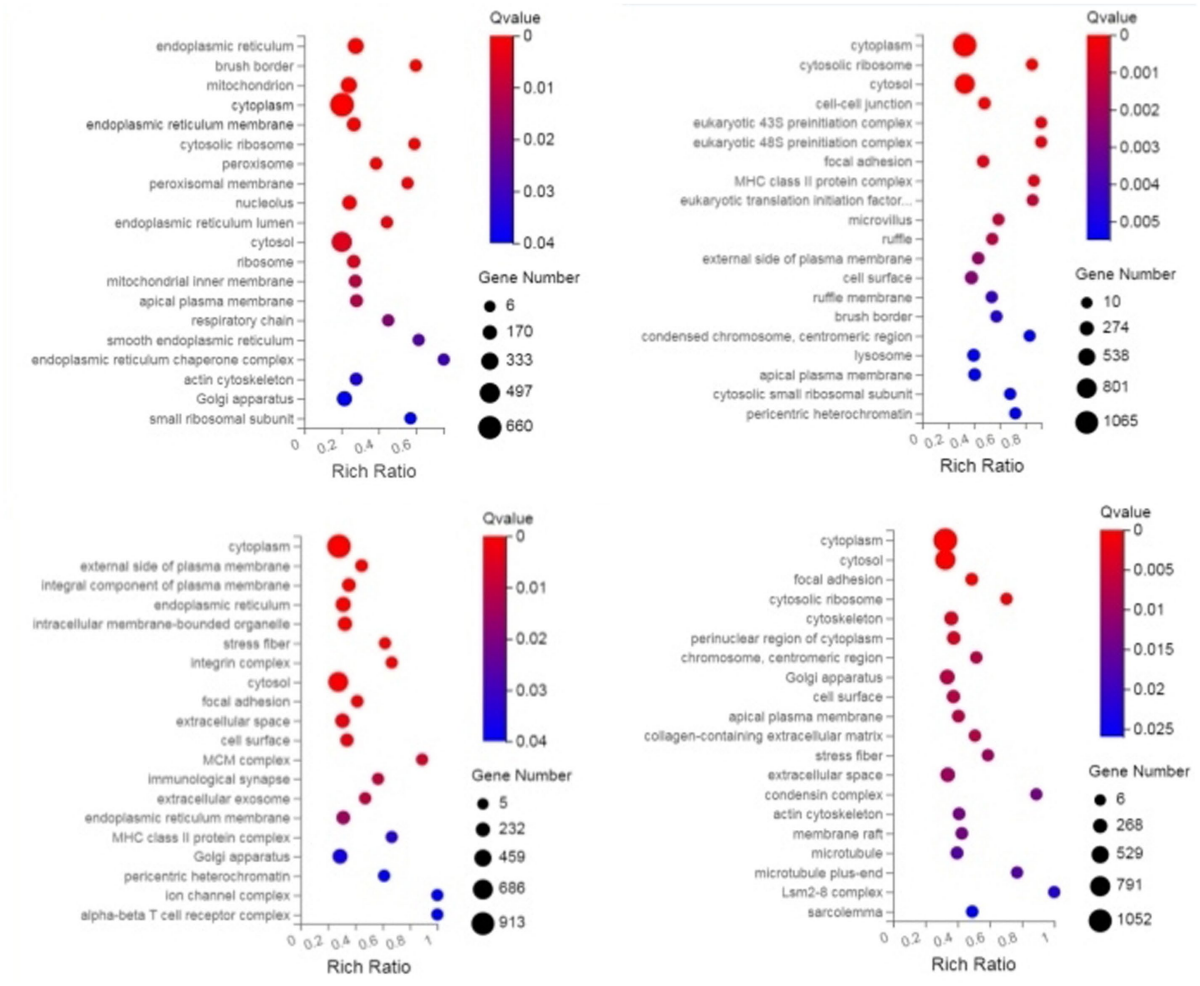
KEGG enrichment bubble plot of DEGS (sequentially infection L3 group, infection L4 group, infection adult group vs. control group).

Through KEGG enrichment analysis, compared with eggs, the Pathways of DEGs (differentially expressed genes) at each developmental stage were mainly enriched in Protein processing in endoplasmic reticulum and Longevity regulating pathway—multiple species/worm, and additionally, in L2 the DEGs were predominantly enriched in Spliceosome, in L4 they were mainly enriched in Neutrophil extracellular trap formation, mRNA surveillance pathway and MAPK signaling pathway, in L5 group the DEGs were primarily enriched in Lysosome and Autophagy—animal, while in the male worm group the DEGs were significantly enriched in Complement and coagulation cascades and Phagosome; these KEGG analyses of DEGs reflect the rapid transcription of genes from the free-living stage to the parasitic stage to adapt to different survival environments (Figure 3).

### 3.4 GO and KEGG enrichment analysis of DEPs

**Figure 4:**
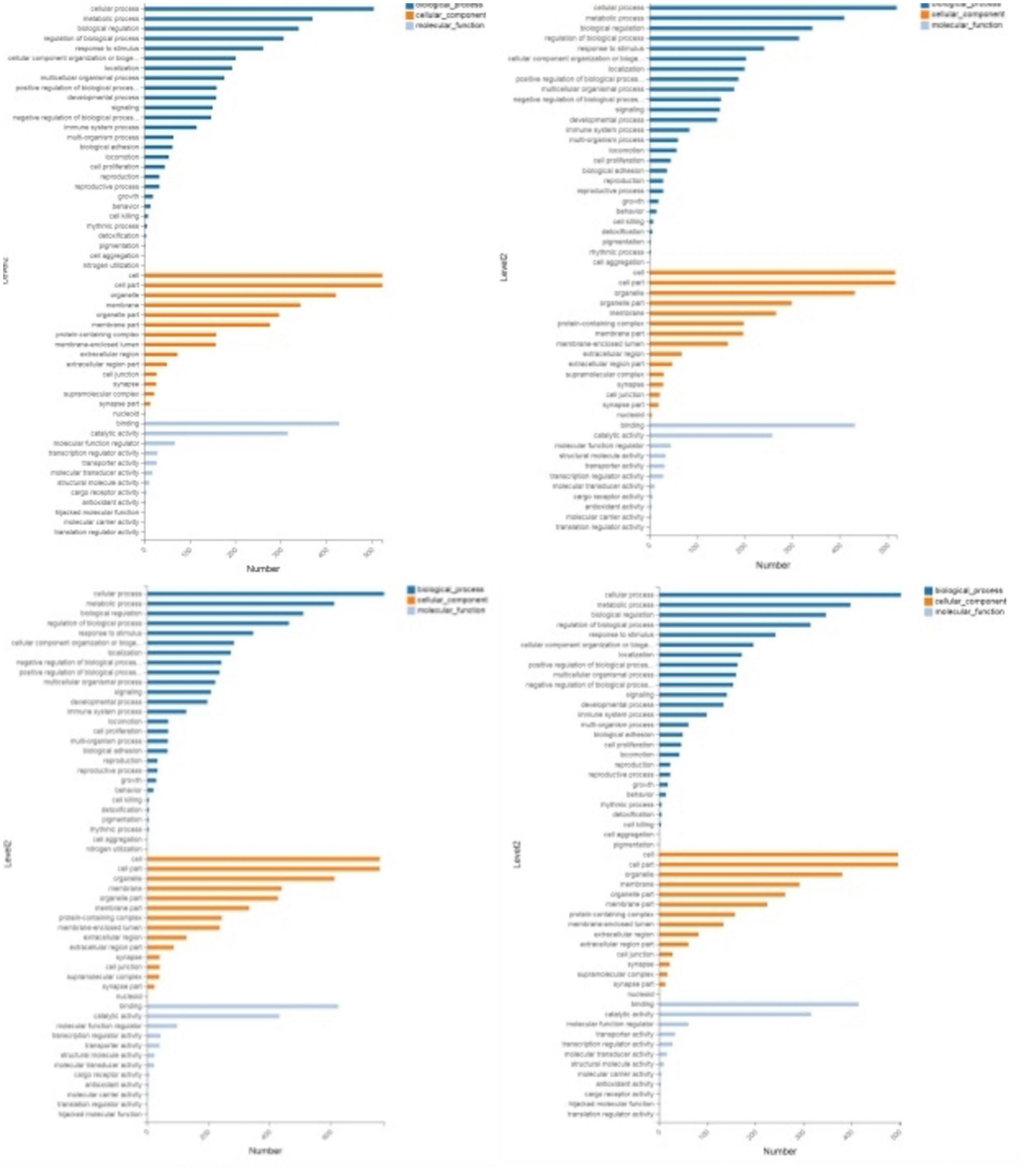
GO enrichment categorization plot of DEPS (sequentially infection L3, infection L4, infection L5 and infection adult vs. control).

**Figure 5:**
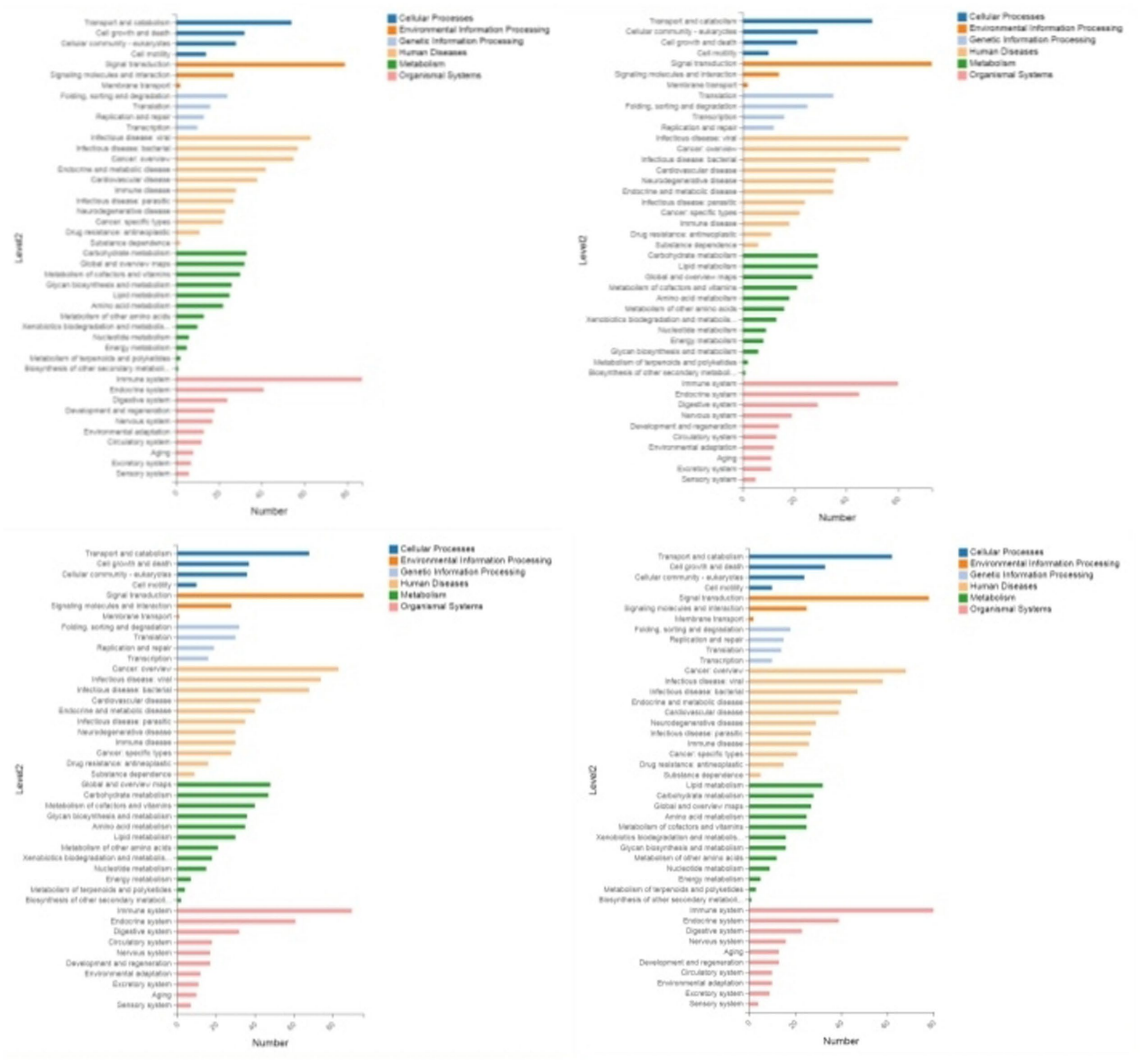
KEGG enrichment categorization plot of DEPS (sequentially infection L3, infection L4, infection L5 and infection adult vs. control).

Through GO enrichment analysis, compared with eggs, DEPs (differentially expressed proteins) at each developmental stage were predominantly enriched in cellular process and metabolic process within biological processes, in cell and cell part within cellular components, and in binding and catalytic activity within molecular function GO terms. Through KEGG enrichment analysis, compared with the control group, DEPs in L1 were predominantly enriched in pathways such as Spliceosome, Focal adhesion, Toxoplasmosis, and Amino sugar and nucleotide sugar metabolism within cellular processes; DEPs in L2 were mainly enriched in pathways including RNA transport, Phagosome, and PPAR signaling pathway; DEPs in L3 were predominantly enriched in Protein processing in endoplasmic reticulum, Phagosome, and Glucagon signaling pathway; DEPs in L4, L5 groups and male worms were all mainly enriched in Metabolic pathways; DEPs in female worms were predominantly enriched in Metabolic pathways and Carbon metabolism. The GO and KEGG enrichment analyses of these DEPs reflect the protein expression differences of *N. oiratianus* from the free-living to parasitic stages, adapting to different survival environments and rapid development (Figures 4, 5).

### 3.5 Results of RT-qPCR

**Figure 6:**
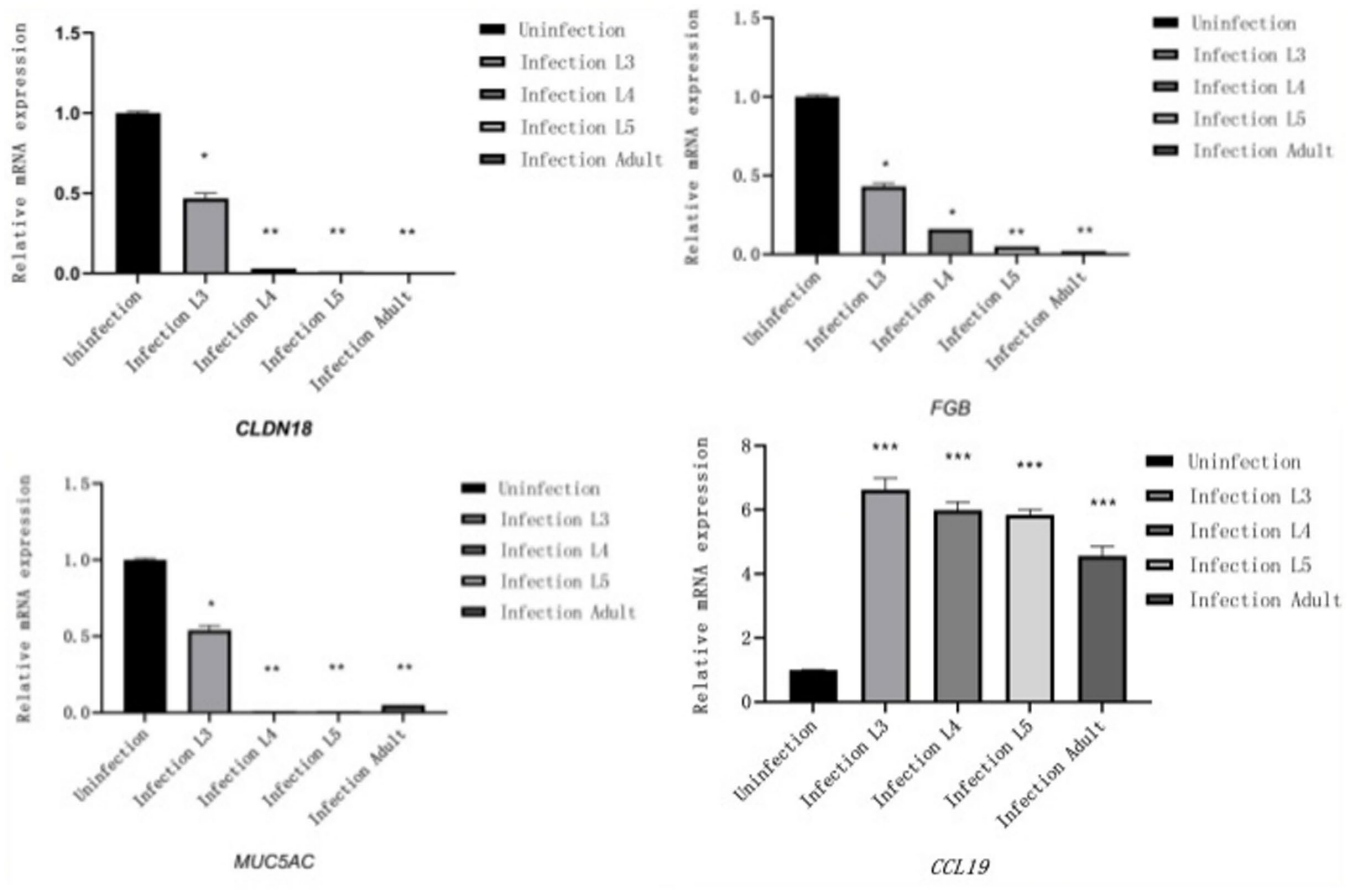
Results of RT-qPCR (The X-axis represents different infection periods, and the Y-axis represents relative expression levels).

Reverse Transcription quantitative PCR (RT-qPCR) was used to validate four stage-specific differentially expressed mRNAs across four infection stages. Results showed that *MUC5AC*, *FGB*, and *CLDN18* were highly expressed only in the control group and L3 infection group, while *CCL19* exhibited higher expression levels in the infection groups. The RT-qPCR results of differentially expressed mRNAs were consistent with those of RNA-Seq, further confirming the reliability of the RNA-Seq findings in this study (Figure 6).

### 3.6 Immunoglobulin and cytokine test results

**Figure 7:**
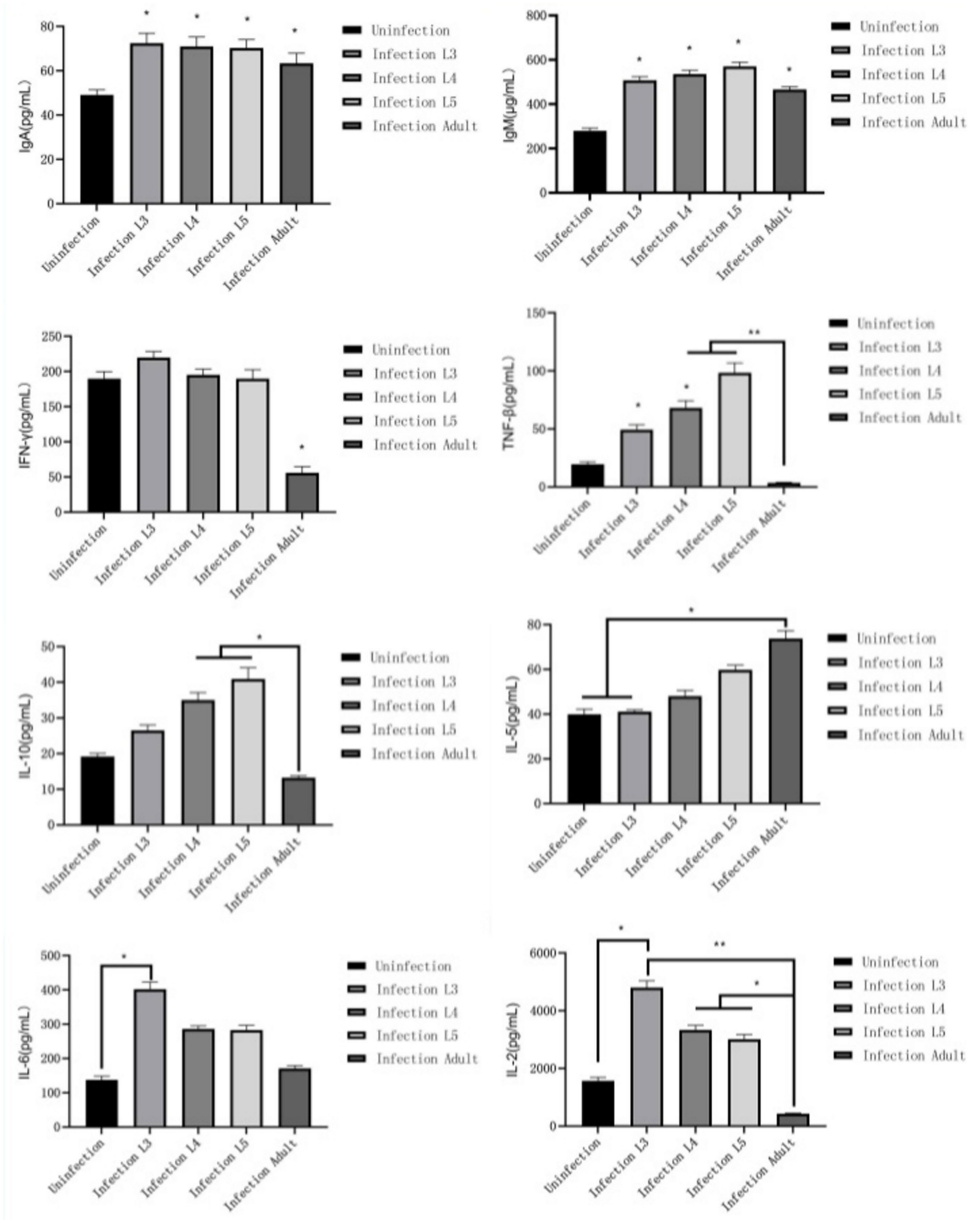
Immunoglobulin and cytokine test results.

After lambs are infected with *N. oiratianus*, the body initially produces specific IgM and IgA in the early stage of infection, with various cytokines all increasing (*p* < 0.05) compared to the uninfected group, thereby resisting *N. oiratianus* infection. However, as the infection duration prolongs, IFN-γ, IL-2, and TNF-β significantly decrease (*p* < 0.05), while IL-5 significantly increases (*p* < 0.05), indicating that Th2-type humoral immunity becomes the main anti-parasitic mechanism (Figure 7).

### 3.7 HE staining and scanning electron microscopy results

**Figure 8:**
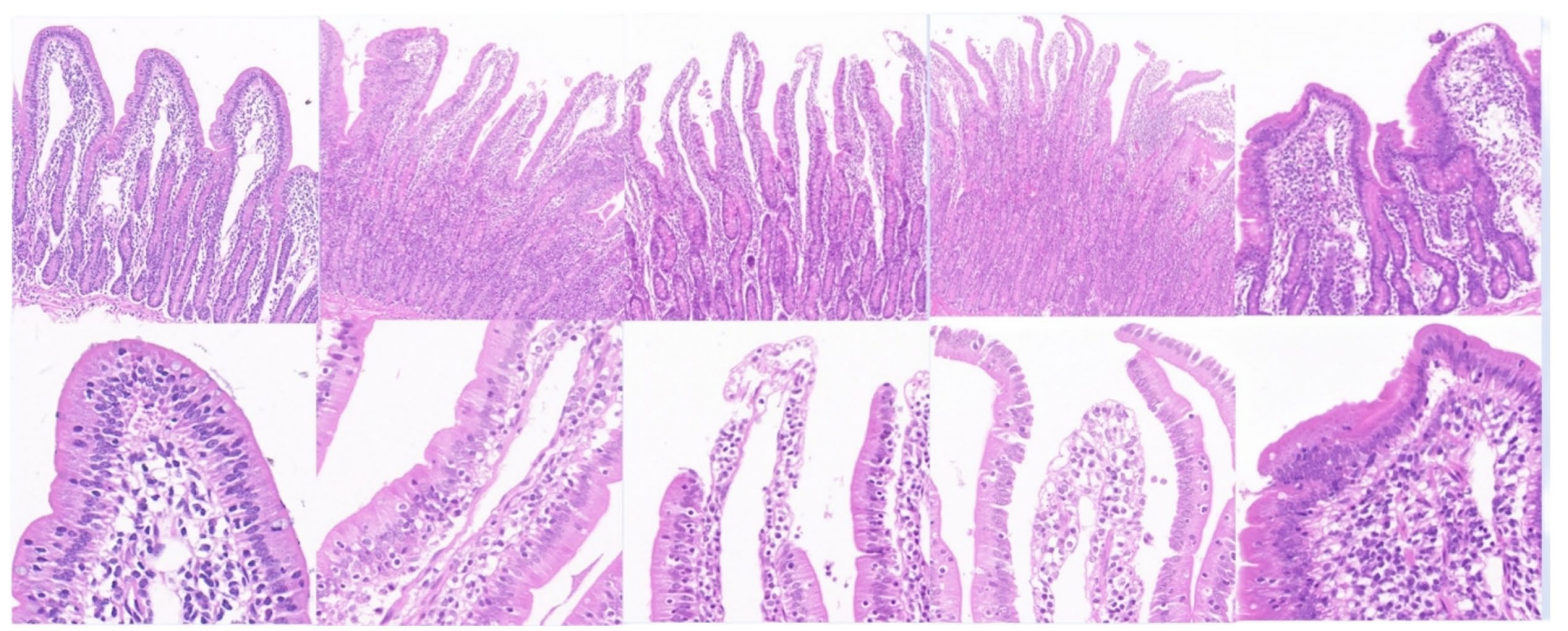
Main tissue changes observed pero endoparasite infection HE staining results (The images are in sequence: uninfected group, infection L3, infection L4, infection L5 and infected adult. The magnification for the first row is 50×, and for the second row, it is 200×).

As shown in Figure 8, the intestinal villi in the control group exhibited a complete structure, with mucosal epithelial cells neatly arranged, goblet cells and striated borders clearly visible, normal lamina propria structure, and intact central lacteals. In the L3 infection group, intestinal villi remained relatively intact, but partial shedding of mucosal epithelial cells from villi was observed, along with edema in the lamina propria and infiltration of numerous mononuclear cells and a small number of eosinophils. In the L4 infection group, intestinal villi were still present, but severe shedding of mucosal epithelial cells occurred, exposing the lamina propria directly to the intestinal lumen, with dilation of central lacteals in the mucosa. In the L5 infection group, intestinal villi were preserved, but severe shedding of mucosal epithelial cells led to direct exposure of the lamina propria to the intestinal lumen, accompanied by edema in the lamina propria. In the adult worm infection group, intestinal villi were swollen due to mononuclear cell infiltration or edema in the lamina propria, with focal shedding of mucosal epithelium. As shown in Table 1, with the progression of infection, the height of intestinal villi significantly decreased (*p* < 0.05), crypt depth significantly increased (*p* < 0.05), and the villus height/crypt depth ratio (V/C ratio) also significantly decreased (*p* < 0.05), indicating a decline in the intestine’s ability to absorb nutrients and an increased probability of diarrhea.

**Table 1:** Height of intestinal villi and depth of crypts at the same stage of infection (μm).

Indicators	Groups				
	Uninfection	Infection L3	Infection L4	Infection L5	Infection adult
Intestinal villus height/μm	643.63 ± 51.6a	526.23 ± 13.47b	381.5 ± 4.69c	364.4 ± 21.72c	267.4 ± 20.25d
Crypt depth/μm	197.57 ± 31.7a	366.6 ± 19.84b	402.85 ± 23.49b	463 ± 20.22c	247.37 ± 23.75d
V/C	3.26 ± 1.63a	1.44 ± 0.68b	0.95 ± 0.2c	0.79 ± 1.07d	1.08 ± 0.85c

The same letter indicates that the difference is not significant (*p* > 0.05) and different letters indicate that the difference is significant (*p* < 0.05).

**Figure 9:**
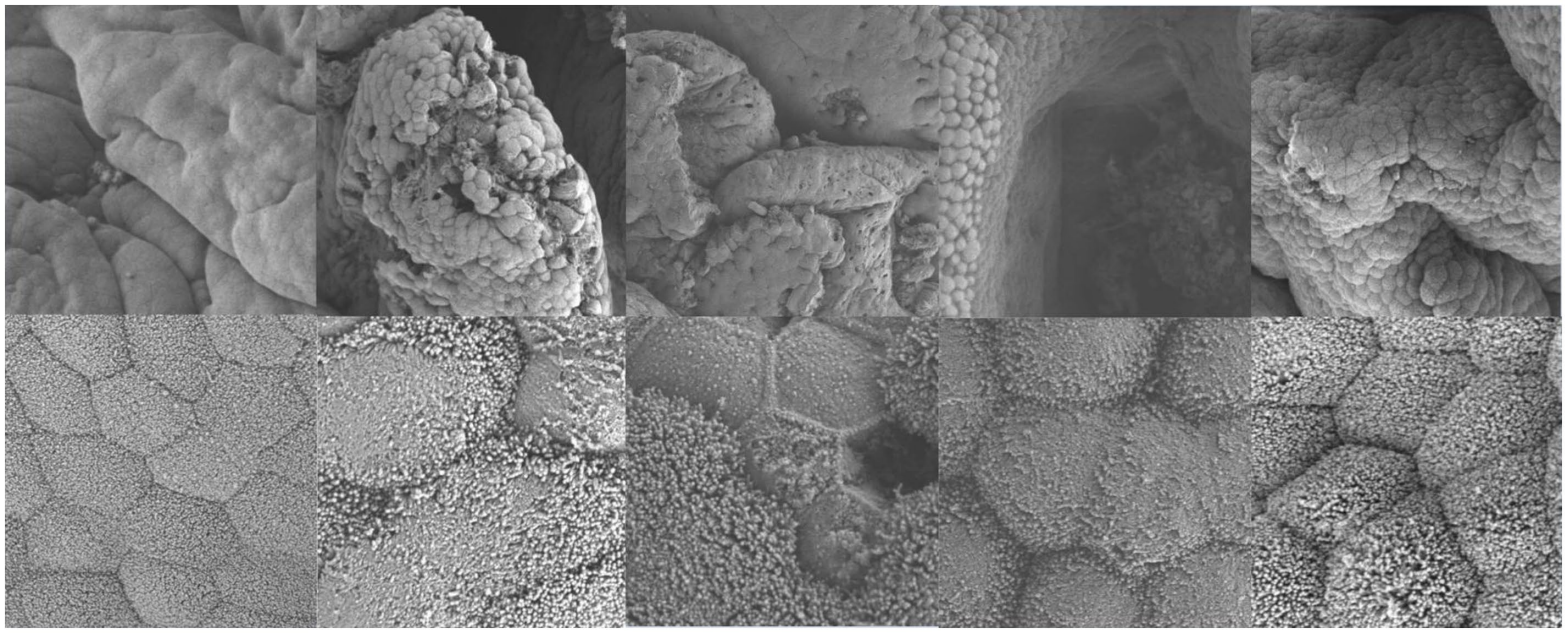
Electron microscopy results (The images are in sequence: uninfected group, infection L3, infection L4, infection L5 and infected adult. The magnification for the first row is 3.0 kv × 3.00 k, and for the second row, it is 3.0 kv × 300 k).

**Figure 10:**
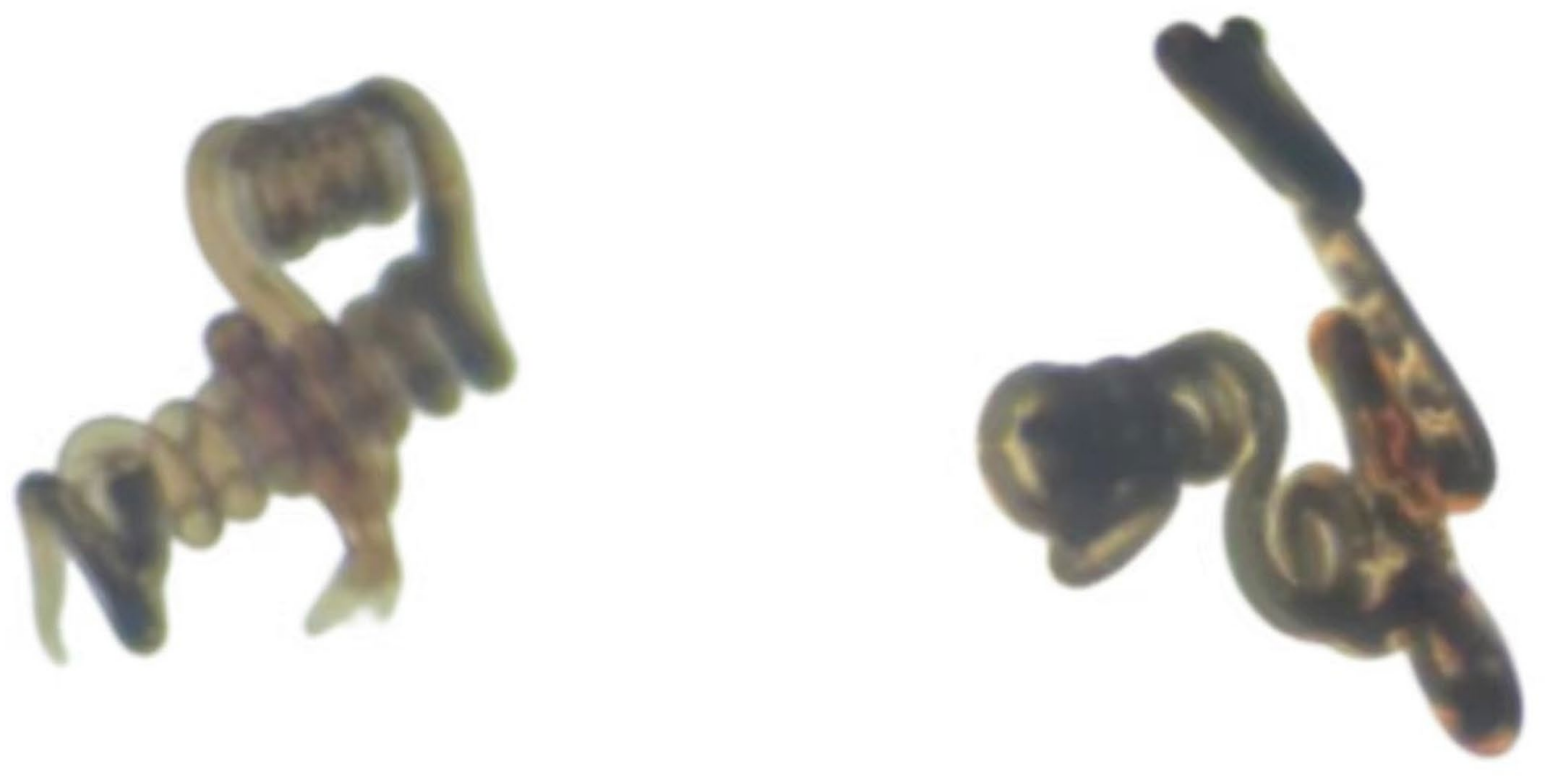
Morphology of *N. oiratianus* during parasitism.

Electron microscopy results showed that in uninfected lambs, intestinal villi were long and slender, with a smooth intestinal wall. After infection with *N. oiratianus*, severe shedding of epithelial cells occurred, and villi became dispersed. The lamina propria swelled due to cell infiltration. With the detachment of the worms, the length of intestinal villi shortened, cell gaps widened, and density decreased significantly (Figure 9). Although electron microscopy did not directly capture the parasitic state of *N. oiratianus*, the newly expelled worms exhibit a helical morphology, indirectly proving that they are not in a free state while parasitizing the intestinal wall. When the worms parasitize, they entwine around the intestinal villi, and the shedding and replacement of intestinal villi may be related to the expulsion (Figure 10).

## 4 Discussion

In many countries, the widespread prevalence of animal infections by nematodes persists, accompanied by significant chronic debilitation post-infection. Presently, effective control of infectious diseases has been achieved through vaccine development and mandatory immunization (30). However, parasitic diseases can only be managed with anthelmintic drugs, leading to severe drug resistance in nematodes due to prolonged and irrational use of anthelmintics (21, 24, 30, 31). This has substantial economic repercussions, particularly in many developing countries and those reliant on livestock farming. The development cycle for new anthelmintic drugs is protracted, while the emergence of resistance to anthelmintics progresses rapidly. Additionally, there is a growing consumer demand for animal-sourced food products free from veterinary drug residues (27, 32). Future strategies for nematode control must rely on sustainable alternative approaches, such as vaccination or selective breeding of nematode-resistant animals, leveraging the host’s natural immune response to nematodes. Currently, the phenomenon of acquired immunity in sheep against *N. oiratianus* provides a valuable model for host resistance against parasitic infections, yet current research remains superficial, lacking precise mechanistic insights.

In this study, through GO and KEGG enrichment analysis, DEGs (Differentially Expressed Genes) and DEPs (Differentially Expressed Proteins) in various groups were significantly enriched in cell–cell junction, phagosome, intestinal immune network for IgA production, chemokine signaling pathway, cell adhesion molecules, immune system, and infectious disease: parasitic pathways. Worms must overcome multiple barriers in the infection process (33), with the first barrier being the intestinal epithelial cells. In the small intestine, epithelial cells have a very short renewal cycle, migrating towards the tip of the villi (34). Intestinal epithelial cells, including Lgr5 + stem cells, Paneth cells, and others, play crucial roles in infection resistance by promoting post-injury repair, producing antimicrobial peptides and defense factors, and maintaining the stability of the intestinal environment (14, 35–37). In the present study, KEGG enrichment analysis and measurements of immunoglobulins and cytokines revealed that sheep control *N. oiratianus* infection in the early stages by activating the immune system, enhancing carbohydrate metabolism, transport, and breakdown to improve nutrient absorption. During early infection, there is an enhancement of tight junctions (TJ) through strengthened signal transduction. *Claudin18* (*CLDN18*), a TJ protein, is significantly downregulated in all infection groups compared to the control group (38). TJ disruption allows fluid leakage through the epithelial barrier, aiding in parasite expulsion. This phenomenon facilitates the rapid transport of parasitic antigens to local immune cells and lymphoid tissues, promoting the entry of immune cells and other effector molecules into the lumen, benefiting the host (39).

As the infection progresses, KEGG analysis revealed significant enrichment in cancer, chemokine signaling pathways, and phagosome pathways. The mechanisms driving the intestinal anti-worm cascade response to infectious larvae are not fully understood, but damaged epithelial cells release various danger signals such as ATP, IL-25, IL-33, and Tslp, driving a Th2-type immune response. The importance of T cells in the anti-worm response was confirmed in classical experiments, showing that in normal hosts infected with worms, initial T cell differentiation into Th2 cells is more critical than Th1-type immune responses (40–43). In the late stages of infection, transcriptional analysis revealed upregulation of chemokine CCL expression, which can influence Th cell polarization (44). The innate and adaptive Th2-type immune responses activated by parasites in epithelial cells mediate a series of cellular and physiological changes at the epithelial cell interface, which greatly impact the survival of parasites. In the late stage of infection, transcriptome KEGG analysis revealed an upregulation of the chemokine factor *CCL*. Chemokine factors of the *CCL* subfamily, such as *CCL19*, *CCL20*, and *CCL21*, can influence Th cell polarization. It has been reported that mice lacking *CCL2* are unable to produce a Th2-type immune response and can only synthesize very low levels of Th2-type cytokines IL-4, IL-5, and IL-10, which may be related to the immune evasion mechanism of nematodes (45, 46). Many parasites acquire energy in the host similar to cancer (47). In the late stage of infection, cancer-related signaling pathways are significantly enriched. In other studies, *Schistosoma* infection has been linked to the occurrence of bladder cancer, as the inflammatory environment caused by parasitic infection creates a precancerous environment (48, 49). The reason parasites can induce cancer after infection may be due to the suppression of immune surveillance, leading to the immune system’s inability to clear certain mutated cells in the host. The expansion of regulatory T cells and the alternative activation of macrophages also create an immune environment favorable to tumor development (50). The impact of parasitic infections on cancer progression requires further attention.

*Muc5ac* is a mucin, a type of glycoprotein normally expressed in non-intestinal mucosa, known to be involved in the inflammatory processes of diseases such as ulcerative colitis and adenocarcinoma. It is co-expressed with *Muc2* in the intestinal tract during inflammation (51). A recent study found an elevation in the transcription levels of *Muc5ac* in bronchial epithelial cells following infection with the Brazilian hookworm in rats (52). IL-13-induced *Muc5ac* is crucial for expelling worms, and the absence of *Muc5ac* (53) in mice has been shown to increase susceptibility to whipworms in comparison to wild-type mice. In contrast to expectations, our findings indicate a significant downregulation of *Muc5ac* at all stages of infection compared to the control group. The distribution of *Muc5ac* in the mucus layer might influence the biochemical properties of the mucus gel, thus promoting the expulsion of nematodes. Additionally, there was a significant downregulation of fibrinogen beta chain (*FGB*) in all groups compared to the control. *FGB* is cleaved by the protease thrombin to produce monomers, which then aggregate with fibrinogen alpha (*FGA*) and fibrinogen gamma (*FGG*) to form insoluble fibrin matrix. Fibrin deposition is also associated with infection (54), and the aggregation of *FGG*, *FGA* and *FGB* forms insoluble fibrin matrix, playing a crucial role in blood clotting (55–57). These observations may represent a potential mechanism by which sheep resist infection by *N. oiratianus*.

Reports suggest that the *N. battus* is believed to utilize villi structures to maintain its position, preventing it from leaving its parasitic site due to peristalsis and movement within the intestine. This study found that the intestinal villi of infected adult groups were shorter compared to the uninfected group. Worms present on the mucosal surface cause damage to villous epithelial cells, leading to more shedding of these cells into the intestinal lumen and resulting in ulcers on the intestinal wall. Additionally, the shape of the villi changes to adapt to the reduced cell numbers. The mechanism by which *N. oiratianus* damages epithelial cells is still unclear. It could be due to direct contact with the parasite or through secretions and excretions of the worm. The reduction in epithelial cells diminishes the absorptive capacity of lambs, and lambs with significant changes in villous structure and reduced mucosal enzyme activity exhibit noticeable diarrhea. In this study, various types of immunoglobulins and cytokines increased in the early stages of infection, with a shift from Th1 to Th2 response, indicating a transition from cellular immunity to humoral immunity to combat worm infection. Epithelial cells and immune cells play significant roles in fighting infection, with infected sheep showing a Th2 immune response. It has also been reported that the *Nematodirus* can form crystals after severe infection, which can block the rectum at the posterior end of the intestine, leading to fluid accumulation post-blockage and resulting in degeneration of reproductive organs (58, 59). Worm infection also reduces the activity of various intestinal enzymes, including aminopeptidases, alkaline phosphatases, and disaccharidases involved in protein and sugar digestion (60, 61). The intestine can also alter the pH of the intestinal lumen, gut microbiota, and control shedding of intestinal villi to resist parasite infection. Various genes and proteins identified in this study, such as the regulation of TJ proteins and differential expression of mucin *Muc5a*c, play crucial roles in worm expulsion and affect the colonization of *N. oiratianus* in the intestine. These genes and proteins identified in this study are particularly important for controlling worm infections and regulating host immune responses. Therefore, they have significant potential for drug or vaccine development.

## 5 Conclusion

*Nematodirus* is a harmful parasite that infects ruminants like sheep, causing economic losses. Overuse of existing anthelmintics has led to drug resistance in nematodes. This study aimed to investigate the mechanism of acquired immunity to *Nematodirus* in sheep by analyzing the genes and proteins in the duodenum of sheep at different stages of infection. RNA-seq and 4D-DIA sequencing were used for the first time to describe the dynamic changes in the duodenum of lambs infected with *N. oiratianus*, and to sequence and analyze the eggs, L1, L2, L3, L4, L5 and male and female worms of *N. oiratianus* for the first time. The study showed that the immune system of lambs was first activated after infection with *N. oiratianus*, and the intestinal epithelial cells played a crucial role in expelling nematodes. Changes in key genes such as *CLDN18*, *CCL19*, and *MUC5AC* may contribute to the expulsion of *N. oiratianus*.

## Data Availability

The data presented in this study are deposited in the NCBI repository, accession number PRJNA1300369.
