# Supplementary material for: Acquired immunity mechanisms in sheep infected with Nematodirus oiratianus: key genes and host responses
Source: Front Vet Sci. 2025 Sep 4;12:1643786. doi: 10.3389/fvets.2025.1643786 (PMC12445057; doi:10.3389/fvets.2025.1643786)
Supplement: Supplementary file 1 [file Supplementary_file_1.docx]

Supplementary Material

# Supplementary Figures and Tables

## Supplementary Figure


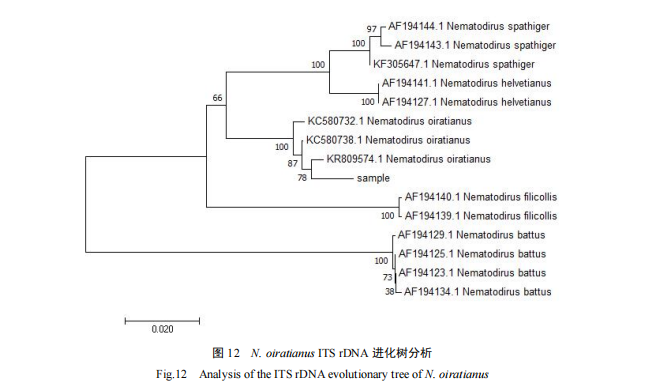


**Supplementary Figure 1.** Analysis of the ITS rDNA evolutionary tree of *N. oiratianus*

## 1.2 Supplementary Table

**Table.1** Primer sequence

| Primer Name | Primer Sequence (5'-3') | Fragment Length（bp） | Annealing Temperature（℃） |  |
| --- | --- | --- | --- | --- |
| *CLDN18*-S | GCACACCACCATGTCTGTGA | 288 | 60 |  |
| *CLDN18*-A | CCAATGACGCTCAGGACGAT |  | 60 |  |
| *CCL19*-S | TGTATTCCTGGTGCGAGCCTA | 193 | 60 |  |
| *CCL19*-A | GGGATGTCATTGGGTAACTGC |  | 60 |  |
| *MUC5AC*-S | TTCATGTGGAACCAGGACGA | 114 | 60 |  |
| *MUC5AC*-A | GAAGAACTCATTGTAGACGGGGAC |  | 60 |  |
| *FGB*-S | CTCGTCCCGTCACAGAAACT | 162 | 60 |  |
| *FGB*-A | CACTCTTTCGGACTGGTCGT |  | 60 |  |
| *gapdh*-S（internal control） | TGGCATCGTGGAGGGACTTA | 266 | 60 |  |
| *gapdh*-A（internal control） | CATCATACTTGGCAGGTTTCTCC |  | 60 |  |
